# Population genetic diversity and structure of *Tephritis angustipennis* and *Campiglossa loewiana* (Diptera: Tephritidae) based on *COI* DNA barcodes in the three-river source region, China

**DOI:** 10.1093/jisesa/ieae075

**Published:** 2024-07-18

**Authors:** Li-Jun Zhang, Ying Liu, Yan-Long Wang, Le-Le Xie, Xin-You Wang, Yu-Shou Ma

**Affiliations:** Qinghai Provincial Key Laboratory of Adaptive Management on Alpine Grassland, Key Laboratory of Superior Forage Germplasm in the Qinghai-Tibetan Plateau, Qinghai Academy of Animal and Veterinary Sciences, Qinghai University, Xining, Qinghai, China; Qinghai Provincial Key Laboratory of Adaptive Management on Alpine Grassland, Key Laboratory of Superior Forage Germplasm in the Qinghai-Tibetan Plateau, Qinghai Academy of Animal and Veterinary Sciences, Qinghai University, Xining, Qinghai, China; Qinghai Provincial Key Laboratory of Adaptive Management on Alpine Grassland, Key Laboratory of Superior Forage Germplasm in the Qinghai-Tibetan Plateau, Qinghai Academy of Animal and Veterinary Sciences, Qinghai University, Xining, Qinghai, China; Qinghai Provincial Key Laboratory of Adaptive Management on Alpine Grassland, Key Laboratory of Superior Forage Germplasm in the Qinghai-Tibetan Plateau, Qinghai Academy of Animal and Veterinary Sciences, Qinghai University, Xining, Qinghai, China; Qinghai Provincial Key Laboratory of Adaptive Management on Alpine Grassland, Key Laboratory of Superior Forage Germplasm in the Qinghai-Tibetan Plateau, Qinghai Academy of Animal and Veterinary Sciences, Qinghai University, Xining, Qinghai, China; Qinghai Provincial Key Laboratory of Adaptive Management on Alpine Grassland, Key Laboratory of Superior Forage Germplasm in the Qinghai-Tibetan Plateau, Qinghai Academy of Animal and Veterinary Sciences, Qinghai University, Xining, Qinghai, China

**Keywords:** *Tephritis angustipennis*, *Campiglossa loewiana*, geographic population, mitochondrial *COI*, genetic diversity, genetic structure

## Abstract

*Tephritis angustipennis* (Diptera: Tephritidae) and *Campiglossa loewiana* (Diptera: Tephritidae) are phytophagous pests in China. Their damage has significantly impacted the collection and cultivation of germplasm resources of native Asteraceae plants. However, the genetic characteristics and structure of their population are unclear. This study focused on the highly damaging species of *T. angustipennis* and *C. loewiana* collected from the three-river source region (TRSR). We amplified the mitochondrial cytochrome C oxidase subunit I (mt*COI*) gene sequences of these pests collected from this area and compared them with *COI* sequences from GenBank. We also analyzed their genetic diversity and structure. In *T. angustipennis*, 5 haplotypes were identified from 5 geographic locations; the genetic differentiation between France population FRPY (from Nylandia, Uusimaa) and China populations GLJZ (from Dehe Longwa Village, Maqin County), GLDR (from Zhique Village, Dari County), and GLMQ (from Rijin Village, Maqin County) was the strongest. GLJZ exhibited strong genetic differentiation from GLDR and GLMQ, with relatively low gene flow. For *C. loewiana*, 11 haplotypes were identified from 5 geographic locations; the genetic differentiation between the Chinese population GLMQ-YY (from Yangyu Forest Farm, Maqin County) and Finnish population FDNL (from Nylandia, Uusimaa) was the strongest, with relatively low gene flow, possibly due to geographical barriers in the Qinghai–Tibet plateau. Only 1 haplotype was identified across GLDR, GLMQ, and GLBM. High gene flow between distant locations indicates that human activities or wind dispersal may facilitate the dispersal of fruit flies and across different geographic. Geostatistical analysis suggested a recent population expansion of these 2 species in TRSR. Our findings provide technical references for identifying pests in the TRSR region and theoretical support for managing resistance, monitoring pest occurrences, analyzing environmental adaptability, and formulating biological control strategies for Tephritidae pests on Asteraceae plants.

## Introduction

Tephritidae, belonging to the order Diptera, are commonly known as fruit flies, which are holometabolous insects. Reproduction typically occurs through oviposition (Ovtshinnikova and Ovchinnikov 2016, Zhang 2019, Korneyev 2021, Mazzon et al. 2021). This family of insects has a large and diverse population with widespread distribution. Currently, more than 4,600 species of Tephritidae have been identified worldwide (Jiang 2015), except from the polar regions (Aluji and Norrbom 1999). Tephritidae have a wide range of hosts, including fruits, inflorescences, and other plant tissues. They typically parasitize subdermal or floral parts as eggs, larvae, pupae, or adult forms (Lutovinovas 2006, Yu 2015, Chen 2017). They are important phytophagous and saprophagous pests of nuts, citrus fruits, vegetables, and Asteraceae, making them prominent seed predators (Korneyev 1996, Merz 2000, Pflugbeil et al. 2021).

In China, more than 570 species of fruit flies are primarily classified into 3 subfamilies (Yu 2015, Chen 2017). Among these, the Tephritinae subfamily exhibits exceptionally high diversity (Yu 2015, Chen 2017). These insects primarily infest Asteraceae plants and engage in complex interactions (Yu 2015, Chen 2017). This study focus on *Tephritis angustipennis* and *Campiglossa loewiana*. The former is also called *Trypeta angustipennis* (synonym) (Heyden 1844), and the latter is also known as *C. loewiana* (Hendel and Lindner 1927) or *Paroxyna loewiana* (synonym). Both species are important Tephritidae pests. Field investigations revealed that these 2 pest species significantly damage the inflorescences of *Aster* in the three-river source region (TRSR). In severe cases, they create cavities within the inflorescences, further preventing the formation or maturation of seeds. Together, these damages lead to poor seed production and cause substantial ecological losses. They pose significant challenges to the collection and utilization of *Aster* genetic resources in the TRSR. Therefore, understanding the classification, life history, and genetic characteristics of these pests is essential. Studies have shown that the eggs, larvae, and pupae of fruit flies are relatively small and exhibit extremely similar morphologies at each stage, making species differentiation difficult based on morphology alone. Due to the high number of closely related species, similar morphologies, and limited research on the classification system of fruit flies in our research area, accurately identifying and classifying species are challenging (Korneyev 2021, Mazzon et al. 2021, Jennings 2023).

New molecular methods to identify, delimit species, and assess community composition, along with species richness in biodiversity and ecological studies, have been developed in recent years. Mitochondrial DNA (mtDNA) genes, such as cytochrome C oxidase subunit I (*COI*), 16S ribosomal RNA (*16S* rRNA), cytochrome oxidase II gene (*COII*), NADH dehydrogenase subunit 2 (*ND2*), and NADH dehydrogenase subunit 5 (*ND5*), have been utilized to study the evolutionary relationships and genetic variation characteristics of 16 orders of insects (Vaňhara et al. 2003). Researchers have combined traditional morphological identification with molecular methods for *COI* to construct phylogenetic relationships between fruit flies and their host plants on the eastern edge of the Qinghai–Tibet Plateau (QTP) (Chen 2017). A study utilized *COI* sequences to investigate the morphological and molecular characteristics of 58 species of Tephritidae from 22 genera and 4 subfamilies (Jiang 2015). Liu (2021) studied the genetic differentiation of *Tephritis femoralis* (Diptera: Tephritidae) mediated by 2 Asteraceae species and the species differentiation of *Pteromalus albipennis* (*Hymenoptera:Pteromalus*) in the Hongyuan area of northwest Sichuan. Therefore, incorporating molecular data alongside morphological identification effectively resolves classification issues among closely related Tephritidae species and delineate their genetic diversity (Roslin et al. 2022, Dominiak et al. 2023).

As the largest family of dicotyledonous plants in the TRSR (Jiao et al. 2021, Wang et al. 2021), Asteraceae are widely distributed across diverse habitats, including marsh meadows, alpine meadows, and alpine cold meadows. The flowering periods of different Asteraceae species continuously span from early to late growing seasons, and their capitulum inflorescence provides an excellent habitat for the larval development of fruit flies. However, many studies on Tephritidae, both domestic and international, have focused on morphology (Jiang 2015, Yaran and Grmez 2020, Yaran and Kutuk 2020, Deghiche-Diab et al. 2021, Korneyev et al. 2021b), occurrence (Zhang 2019, Harym et al. 2020, Deghiche-Diab et al. 2021, Korneyev et al. 2021a, 2021b, Nilsson et al. 2022), biology (Wang 1990a, 1990b, Han 2019, Korneyev and Namin 2019, Namin 2019, Evstigneev and Glukhova 2020, Freidberg et al. 2020, Gaimari and Silva 2020), phenological synchrony with hosts and phylogenetic relationships (Chen 2017, Kütük et al. 2018, Korneyev et al. 2020, Morgulis et al. 2020), geographic distribution patterns (Yu 2015, Evstigneev and Korneyev 2018, Korneyev and Korneyev 2018, Rull et al. 2019, Korneyev et al. 2021a), and control methods (Reinert et al. 2020, Ao et al. 2023, Chen et al. 2023, Gong et al. 2023, Lu et al. 2023, Mu 2023, Quan 2023, Xu et al. 2023, Zhou et al. 2023). Domestic research primarily addresses the distribution and genetic differentiation of common quarantine Tephritidae, such as *Bactrocera minax* (Ouyang et al. 2021, Jia et al. 2023) and *Bactrocera dorsalis*, or invasive agriculture and forestry pests (Zhao et al. 2019, Cui et al. 2020, Chen et al. 2021, Shao et al. 2022). The genetic structure and variation of Tephritidae populations severely affect the ripening of *Aster* seeds in the TRSR. This interaction may be shaped by complex factors such as genetic adaptability, ecological interactions, genetic drift, and environmental adaptability (Abrosov and Bogolyubov 1985, Sapp et al. 2020). The outbreak of both pests on *Aster* in the TRSR may be related to the combined effects of these factors, which likely determine the interaction patterns and outcomes between fruit fly populations and host plants in specific ecological environments. However, whether these factors collectively contribute to the adaptation of fruit fly populations to specific habitats and host plants remains unexplored. Moreover, local specimens of Tephritidae are scarce, and research on this family in the TRSR is limited, particularly concerning classification, distribution, and systematic development. And studies on genetic evolution and relationships between 2 species populations in this area and other countries are unclear.

We selected adult’s specimens of 2 Tephritidae species that severely damaging the capitulum of *Aster* inflorescence, collected from the wild, for morphological observations. Based on the *COI* barcoded data, the genetic diversity, and phylogenetic relationships of *T. angustipennis* and *C. loewiana* in the TRSR were explored. The genetic or haplotype characteristics of these 2 pests and their systematic developmental relationships and genetic characteristics were compared with published mt*COI* sequences of *T. angustipennis* and *C. loewiana*, revealing evolutionary relationships at the molecular level. The aims of this study were to provide a basis for the accurate classification and identification of these pests and to understand succession patterns for effective control of *Aster* species in the TRSR.

## Materials and Methods

### Specimen Collection

**Fig. 1.**
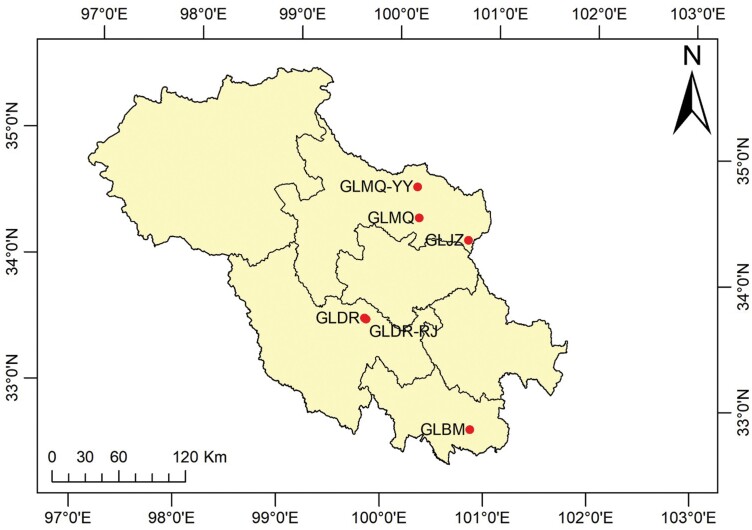
Sample collection sites different geographical populations of *T. angustipennis* and *C. loewiana* in the three-river source region (TRSR), China. After investigation, the 2 pests were not found in other areas of the TRSR, so only the locations where the data had been collected were marked in the figure. Populations sequences data for FDNY, FRPY, FDNL, and GETU were obtained from NCBI and not marked in the figure. Sample plot by Li-Jun Zhang and Xin-You Wang.

Samples of *T. angustipennis* and *C. loewiana* were collected from the flowers of *Aster farreri* (Asteraceae: *Aster*), *Aster diplostephioides* (Asteraceae: *Aster*), *Aster poliothamnus* (Asteraceae: *Aster*), and *Aster souliei* (Asteraceae: *Aster*) in Guoluo Prefecture, Qinghai Province, between July and October 2023. Adult flies were collected using sweeping nets and aspirators, and sampling information was recorded. The specimens were temporarily preserved in 75% ethanol, classified, and identified indoors. Adult flies collected from the field were used for DNA extraction. Basic information, including collection location, collection time, and distribution of each population is provided in Table 1 and Fig. 1, and sampling plots were labeled using ArcGIS (V10.8). Voucher specimens are stored in the laboratory of Qinghai Academy of Animal and Veterinary Sciences, Qinghai University (voucher numbers: MQ20230804AF3 and MQ20230918AF4). In addition to the 16 *T. angustipennis* (sample numbers: 1, 7, 11–13, 23–28, 34–38) and 11 *C. loewiana* (sample numbers: 15–22, 29, 33, 42) self-collected individual mt*COI* sequences, the remaining were downloaded from the GenBank database. The method of *T. angustipennis* search from the *COI* barcode database involved the keyword “*Tephritis*” + “*COI*.” The keywords for *C. loewiana* were “*Campiglossa*” + “*COI*.” All *COI* sequences found from the search were downloaded, and detailed information such as NCBI accession numbers can be found in Supplementary Table S1. Only 3 submitted *T. angustipennis COI* sequences were available in NCBI from the 7 submitted sequences of *C. loewiana*. All downloaded sequences were used for phylogenetic analyses.

**Table 1.** Sample information of different geographical populations of *T. angustipennis* and *C. loewiana*

No.	Collecting locality	Populations code	Host plant	Latitude and longitude	Attitude (*m*)	Collection date	Sample size	Sequence size	PCR success rate (%)
1	Nylandia, Uusimaa, Finland	FDNY	N/A	N/A	N/A	N/A	2	2	100.00
2	Pyrenees Orientals, Mont Louis, La Llagonne, France	FRPY	*Achillea ptarmica*	E: 00°39ʹ28ʹʹ, N: 42°37ʹ56ʹʹ	3,404.00	2011.06.20	1	1	100.00
3	Dehe Longwa Village, Maqin County, Guoluo Prefecture, Qinghai Province, China	GLJZ	*Aster farreri*	E: 100°76ʹ25ʹʹ, N: 34°31ʹ18ʹʹ	3,782.40	2023.07.04	14	5	35.71
4	Rijin Village, Maqin County, Guoluo Prefecture, Qinghai Province, China	GLMQ	*Aster diplostephioides*	E: 100°26ʹ25ʹʹ, N: 34°47ʹ34ʹʹ	3,804.70	2023.09.18	5	6	120.00
5	Zhique Village, Dari County, Guoluo prefecture, Qinghai Province, China	GLDR	*A. farreri*	E: 99°77ʹ38ʹʹ , N: 33°65ʹ43ʹʹ	3,737.60	2023.08.05	6	5	83.33
6	Yangyu Forest Farm, Maqin County, Guoluo Prefecture, Qinghai Province, China	GLMQ-YY	*Aster poliothamnus*	E: 100°23ʹ37ʹʹ, N: 34°71ʹ97ʹʹ	3,492.90	2023.08.12	8	8	100.00
7	Zhique Village, Dari County, Guoluo prefecture, Qinghai Province, China	GLDR-RJ	*Aster souliei*	E: 99°72ʹ02ʹʹ, N: 33°66ʹ40ʹʹ	3,492.90	2023.09.20	5	2	40.00
8	Red Army Ditch, Banma County, Guoluo Prefecture, Qinghai Province, China	GLBM	*A. farreri*	E: 100°85ʹ59ʹʹ, N: 32°80ʹ72ʹʹ	4,203.10	2023.08.08	5	1	20.00
9	Nylandia, Uusimaa, Finland	FDNL	N/A	N/A	N/A	N/A	4	4	100.00
10	Tuv Prov., Tusgalt Valley, Forestry Research-Training Center, Germany	GETU	N/A	E: 106°51ʹ11ʹʹ, N: 48°15ʹ37ʹʹ	1,522.00	N/A	3	3	100.00
	Total						53	37	69.81

“N/A” means missing value, it indicates that specific information about the species is not mentioned or unknown in the reference article. Information was collected from 1 to 5 samples of *T. angustipennis* and from 6 to 10 samples of *C. loewiana*.

### PCR Amplification of a *COI* Fragment and Sequencing

#### DNA Extraction

Twenty-five specimens of *T. angustipennis* and 18 specimens of *C. loewiana* were collected. After morphological identification, insect abdomens were dissected and washed with deionized water to remove surface impurities. Clean samples were placed on clean filter paper to dry. Dehydrated tissues were ground in an ice bath, and total DNA was extracted using the Rapid Animal Genomic DNA Isolation Kit (Sangon Biotech Co., Ltd, Shanghai, China) following the manufacturer’s intructions.

#### PCR Amplification and Sequencing

The primers used for amplifying the *COI* gene of 2 species in this study were universal *COI* primers with the following sequences: LCO1490: 5ʹ-GGTCAACAAATCATAAAGATATTGG-3ʹ and HCO2198: 5ʹ-TAAACTTCAGGGTGACCAAAAAATCA-3ʹ (Tamura et al. 2011). The 25 μl PCR reaction system comprised 2 μl of DNA template, 12.5 μl Taq PCR Master Mix (Sangon Biotech Co., Ltd), 1 μl each of forward and reverse primers, and 8.5 μl of sterile deionized water. The PCR amplification procedure was as follows: 94 °C for 4 min, 35 cycles of 94 °C for 30 s, 48 °C for 30 s, and 72 °C for 50 s, and a final extension at 72 °C for 10 min, with an infinite hold at 16 °C. Subsequently, 2 μl of the PCR amplification product was used for electrophoresis, and PCR products with correct fragment sizes were sent to Shanghai Shenggong Biology Company for bidirectional sequencing. Nine *T. angustipennis* and 7 *C. loewiana* individuals failed to expand.

### Data Analysis

#### Sequence Trimming

First, Seqman software was used to assemble the forward and reverse gene sequences of all samples, and correct unclear sites by observing sequencing peak maps. The assembled sequences were saved in the FASTA format. Assembled sequences were manually proofread with the homologous sequences from NCBI using MEGA X (Kumar et al. 2018). Subsequently, the obtained target sequences were aligned and trimmed using DNAMAN and MEGA X. Primers from both ends were removed, and finally, a complete amplification sequence of 658 bp was obtained. Finally, the sequence accuracy was validated by translating (Wang 2022). The complete sequence was compared using NCBI BLAST, leading to the determination of the species as *T. angustipennis* and *C. loewiana* based on the similarity with the aligned sequences, consistent with the morphological identification. The empty base sequences at the 5ʹ and 3ʹ ends were trimmed to enhance homology and facilitate analysis. Finally, 37 sequences of 645 bp in length segment from the mtDNA *COI* gene of the 2 species were used for subsequent analysis.

#### Barcode Sequence Feature Analysis and Phylogenetic Analysis

*COI* sequences were aligned using CLUSTALW in MEGA X (Kumar et al. 2018), with default parameters set for DNA weight matrix, gap opening, and extension penalties. MEGA X was used for nucleotide diversity analysis and multiple alignment of the obtained sequences. The number of translocation points, specific translocation points, translocation ratio, base composition, and genetic distances were calculated (Meng et al. 2022). Intraspecies genetic distance and interspecific genetic distance of each geographic population were calculated using the Kimura 2-parameter (K2P) genetic distance model (Kimura 1980). A neighbor-joining (NJ) phylogenetic tree was constructed based on the minimum evolutionary distance of the *COI* sequence. Bootstrapping was performed 1,000 times to test the confidence of each branch. Information on reference species and GenBank accession numbers used for evolutionary analysis and phylogenetic tree construction is shown in Supplementary Table S1. The MEME software was used to calculate conserved sites.

#### Genetic Diversity Analysis of Mitochondrial *COI* Gene in Two Species

DnaSPv6.0 (Rozas et al. 2017) was used to analyze the genetic diversity of the sequences, and Tajima’s *D* and Fu’s *F*_*s*_ neutrality tests were performed. The number of haplotypes (*h*) and haplotype diversity (*Hd*) were calculated. Variable sites, separation sites (*S*), nucleotide diversity (*π*), average number of nucleotide differences (*K*), and other genetic diversity parameters were determined (Wang 2022, Liu 2023). The analysis of molecular variance (AMOVA) was analyzed using Arlequin 3.5 (Excoffier and Lischer 2010), and parameters such as interpopulation genetic differentiation index (*F*_*st*_) and gene flow (*N*_*m*_) were calculated. The *F*_*st*_ and *N*_*m*_ of the total population were obtained by DnaSP 6.0 analysis. Network 5.0 (Bandelt et al. 1999) was used to draw the haplotype median-joining network, which was collated in PowerPoint.

## Results and Analysis

### Mitochondrial *COI* Gene Sequence Analysis of Two Species

The average contents of *T*, *C*, *A*, and *G* in all sequences of *T. angustipennis* were 40.33%, 14.41%, 29.72%, and 15.54%, respectively (Supplementary Table S2). The proportions of these bases differed slightly among different geographic populations, but these differences were not statistically significant, and the base composition remained stable. The average *A* + *T* content was 70.05%, significantly higher than of the *G* + *C* content (29.95%), indicating base bias. The sequence exhibited 615 conserved sites, 30 mutated sites, 30 mutations, and no insertion/deletion sites. Mutations accounted for 4.65% of the total sequence. Six singleton sites and 24 parsimony-informative sites were identified (Supplementary Table S3). All sequences contained 4 transitions and 2 transversions (Supplementary Table S4).

For *C. loewiana*, the average *A* + *T* content (68.16%) was lower than that of *T. angustipennis* but still much higher than the *G* + *C* content, showing a clear *A* + *T* bias. The contents of *A* and *T* were equivalent (Supplementary Table S2). The sequence showed 610 conserved sites, 35 mutated sites, 35 mutations, and no insertion/deletion sites; mutations accounted for 5.43% of the total sequence. There were 13 singleton sites and 22 parsimony-informative sites (Supplementary Table S3). The number of transitional sites (6) and transversional sites (3) was higher in *C. loewiana* than in *T. angustipennis*, and the conversion-to-transmutation ratio (*R*) of both pests was 2 (Supplementary Table S4). The frequency of conversion was different across populations of *C. loewiana*, with GLMQ-RJ being the most common. The frequency of GLMQ-YY and GETU populations was small, and no transitional and transversional events occurred in FDNL populations. Base conversion types were (*C*→*T*), followed by (*T*→*C*) and (*G*→*A*). The mutational loci of both pests involved 2 variants, with no 3 and 4-base variants.

### Genetic Diversity Analysis

Five haplotypes were identified in the mtDNA *COI* sequence of 19 *T. angustipennis* individuals, with only 1 shared Haplotype (H1) distributed across 2 populations. The H1 haplotype appeared 11 times, accounting for 57.89% of all detected individuals. All others were exclusive haplotypes (H2–H5), present in a single population. The number of polymorphic sites in the *COI* gene sequences ranged from 0 to 1 (Table 2), and the haplotype diversity of all groups was relatively low, with the haplotype diversity index ranging from 0 to 1.00000 and the nucleotide diversity index ranging from 0 to 0.00155. The remaining locations had low values of *Hd* and *Pi*, indicating possible bottleneck effects in the population history.

**Table 2.** Genetic diversity analysis and demographic parameters of mtDNA *COI* gene among 10 different geographic populations of *T. angustipennis* and *C. loewiana*

Populations	*S*	*h*	Haplotypes (number of individuals)	Variance of haplotype diversity	*Hd* ± SD	*π* ± SD	*K*	Tajima’s *D*	Fu’s *F*_*s*_
FDNY	1	2	H3(1), H4(1)	0.25000	1.000 ± 0.500	0.00155 ± 0.00078	1.0000	N/A	N/A
FRPY	N/A	N/A	H5(1)	0.00000	0.00000	0.00000	0.0000	N/A	N/A
GLJZ	0	1	H2(5)	0.00000	0.000	0.00000	0.0000	N/A	0.000
GLDR	1	1	H1(6)	0.03856	0.000	0.00000	0.2860	−1.00623	−0.095
GLMQ	0	1	H1(5)	0.00000	0.000	0.00000	0.0000	N/A	0.000
Total of *T. angustipennis*	30	5	H1(11), H2(5), H3(1), H4(1), H5(1)	0.00984	0.620 ± 0.099	0.01039 ± 0.00243	6.7020	−0.86939	5.227
GLMQ-YY	4	4	H7(1), H9(5), H10(1), H11(1)	0.03390	0.643 ± 0.184	0.00155 ± 0.00120	1.0000	−1.53470	−1.236
GLMQ-RJ	12	2	H8(1), H12(1)	0.25000	1.000 ± 0.500	0.01860 ± 0.00537	12.0000	N/A	N/A
GLBM	N/A	N/A	H6(1)	0.00000	0.00000	0.00000	0.0000	N/A	N/A
FDNL	1	2	H13(3), H14(1)	0.07031	0.500 ± 0.265	0.00078 ± 0.00085	0.5000	−0.61237	0.172
GETU	2	2	H15(2), H16(1)	0.09877	0.667 ± 0.314	0.00207 ± 0.00146	1.3330	N/A	N/A
Total of *C. loewiana*	35	11	H6(1), H7(1), H8(1), H9(5), H10(1), H11(1), H12(1), H13(3), H14(1), H15(2), H16(1)	0.00261	0.908 ± 0.051	0.01366 ± 0.00267	8.8100	−0.54369	−0.285

*Hd*, haplotype diversity; *π*, nucleotide diversity; *K,* average number of nucleotide difference; *h,* number of haplotypes; *S,* number of variable sites; SD, standard deviation. “N/A” means missing value, it also indicates that there are no polymorphisms in the data that cannot be calculated by Tajima’s test.

In contrast, 11 haplotypes (H6–H16) were detected in 18 samples of *C. loewiana* mtDNA *COI* sequences, with no shared haplotypes. H9 and H13 were the dominant haplotypes, with H9 appearing 5 times (27.78%) and H13 appearing 3 times (16.67%), together accounting for 44.45% of all individuals. The *Hd*, *S*, *π*, and *K* values for the 5 populations were 0.908, 35, 0.01366, and 8.8100, respectively (Table 2). The number of polymorphic sites in the *COI* gene sequences ranged from 1 to 12, and the haplotype diversity index ranged from 0.5 to 1.00000, with the nucleotide diversity index ranging from 0.00078 to 0.01860. The GLMQ-RJ population exhibited the highest *Hd*, *π*, and *K* values. And this population has the most polymorphism loci, suggesting that these populations may have experienced a bottleneck followed by rapid growth and mutations accumulation.

### Genetic Differentiation and Molecular Variation Analysis of Two Species

The fixation index (*F*_*st*_) is typically used to analyze the degree of differentiation between populations, while gene flow (*N*_*m*_) indicates the level of gene exchange and the phenomena affecting genetic differentiation. The overall genetic differentiation (*F*_*st*_) for the *T. angustipennis* population was 0.73226, with *F*_*st*_ values between populations ranging from 0 to 1.00000 (Table 3). Genetic differentiation was lowest between GLDR and GLMQ populations (*F*_*st*_ = 0) and highest between the FRPY and GLJZ, GLDR and GLMQ, and between GLJZ and GLDR, GLMQ populations (*F*_*st*_ = 1.00000). The total gene flow (*N*_*m*_) among the 5 populations was 0.18, and the *N*_*m*_ variation ranged from 0 to infinity, indicating a high level of gene exchange among different geographic populations, especially the obvious genetic differentiation among different geographic regions, except for GLDR and GLMQ populations of *C. loewiana.* AMOVA showed (Table 4) that the percentage of genetic variation within populations was 0.84%, and that between populations was 99.16%. The percentage of genetic variation within populations was significantly smaller than that between populations (*P* < 0.01), indicating that variations among populations were the main factor for overall population variation; that is, genetic variation was caused by external factors.

**Table 3.** Pairwise *F*_*st*_ (below the diagonal) and gene flow *N*_*m*_ (above the diagonal) values of geographical populations of *T. angustipennis* and *C. loewiana*

Geographic population	FDNY	FRPY	GLJZ	GLDR	GLMQ	GLMQ-YY	GLMQ-RJ	GLBM	FDNL	GETU
FDNY		0.03226	0.00431	0.00344	0.00414	0.00680	0.04392	0.00719	0.00440	0.00869
FRPY	0.93939		0.00000	0.00000	0.00000	0.00685	0.09231	0.00000	0.00353	0.00939
GLJZ	0.99145	1.00000		0.00000	0.00000	0.00411	0.01565	0.00000	0.00139	0.00291
GLDR	0.99316	1.00000	1.00000		inf	0.00373	0.01319	0.00000	0.00120	0.00246
GLMQ	0.99180	1.00000	1.00000	0.00000		0.00407	0.01586	0.00000	0.00137	0.00287
GLMQ-YY	0.98658	0.98649	0.99185	0.99260	0.99193		0.11103	0.06667	0.04740	0.06970
GLMQ-RJ	0.91925	0.84416	0.96964	0.97430	0.96025	0.81829		0.54545	0.08524	0.13693
GLBM	0.98582	1.00000	1.00000	1.00000	1.00000	0.88235	0.47826		0.05263	inf
FDNL	0.99128	0.99298	0.99723	0.99760	0.99726	0.91340	0.85435	0.90476		0.08850
GETU	0.98291	0.98216	0.99422	0.99511	0.99429	0.87766	0.78502	0.00000	0.84962	

**Table 4.** Analysis of molecular variance (AMOVA) of 10 geographic populations of *T. angustipennis* and *C. loewiana*

Species	Source of variation	*df*	Sum of squares	Variance components	Percentage of variation	Fixation indices
*T. angustipennis*	Among populations	4	59.816	4.19921 Va	99.16%	*F*_*st*_ = 0.73226^**^
	Within populations	14	0.500	0.03571 Vb	0.84%	
	Total variance	18	60.316	4.23492	100%	
*C. loewiana*	Among populations	4	63.306	4.67542 Va	83.99%	*F*_*st*_ = 0.53507^**^
	Within populations	13	11.583	0.89103 Vb	16.01%	
	Total variance	17	74.889	5.56644	100%	

Va and Vb, number of variance components; *df*, degrees of freedom.

**P < 0.01.

### Population Genetic Distance Analysis

The genetic distance among the 5 haplotypes of the mt*COI* gene of *T. angustipennis* ranged from 0.0098 to 0.2724 (Table 5), with an average of 0.1765. The genetic distance between H2 and other haplotypes was notably high, ranging from 0.1764 to 0.2591. The genetic distance across the 5 geographic populations of this species was small, ranging from 0 to 0.0391 (Table 6), with an average of 0.0229. Specifically, the genetic relationship between the GLMQ and GLJZ populations from Maqin County and the GLDR populations from Dairi County was close, ranging from 0 to 0.0016, all at the intraspecific level (Hebert et al. 2003). The difference in the genetic distance between these 3 populations and the other populations was large, indicating a distant genetic relationship between these 3 and the 2 populations outside but within the TRSR is far.

**Table 5.** Genetic distance among 16 haplotypes of *T. angustipennis* and *C. loewiana*

Populations	Hap1	Hap2	Hap3	Hap4	Hap5	Hap6	Hap7	Hap8	Hap9	Hap10	Hap11	Hap12	Hap13	Hap14	Hap15	Hap16
Hap1																
Hap2	0.0098															
Hap3	0.2572	0.2441														
Hap4	0.2724	0.2591	0.0098													
Hap5	0.1885	0.1764	0.1670	0.1803												
Hap6	1.3696	1.3147	1.3381	1.4097	1.3518											
Hap7	1.1890	1.1453	1.2261	1.2850	1.2355	0.0724										
Hap8	1.0953	1.0564	1.0843	1.1298	1.3918	0.2998	0.2000									
Hap9	1.1890	1.1453	1.2261	1.2850	1.2355	0.0724	0.0000	0.2000								
Hap10	1.1364	1.0953	1.1735	1.2261	1.1799	0.0951	0.0198	0.1744	0.0198							
Hap11	1.1890	1.1453	1.2261	1.2850	1.2355	0.0836	0.0098	0.2134	0.0098	0.0300						
Hap12	1.1453	1.1041	1.1298	1.1799	1.1890	0.1877	0.1040	0.1270	0.1040	0.1040	0.1152					
Hap13	1.3696	1.3147	1.3381	1.2756	1.3518	0.0511	0.0836	0.3159	0.0836	0.1069	0.0951	0.2008				
Hap14	1.4289	1.3696	1.3954	1.3286	1.4097	0.0614	0.0943	0.3303	0.0943	0.1178	0.1059	0.2134	0.0098			
Hap15	1.3918	1.3696	1.3954	1.4725	1.4097	0.0098	0.0829	0.3139	0.0829	0.1059	0.0943	0.2000	0.0614	0.0718		
Hap16	1.4531	1.3918	1.4097	1.4932	1.4289	0.0098	0.0614	0.2841	0.0614	0.0836	0.0724	0.1748	0.0404	0.0506	0.0197	

**Table 6.** The mean genetic distance among different geographical populations of *T. angustipennis* and *C. loewiana*

Population	GLMQ	GLJZ	GLDR	FDNY	FRPY	GLBM	GLMQ-YY	GLMQ-RJ	FDNL	GETU
GLMQ										
GLJZ	0.0016									
GLDR	0.0000	0.0016								
FDNY	0.0391	0.0375	0.0391							
FRPY	0.0285	0.0269	0.0285	0.0262						
GLBM	0.1302	0.1284	0.1302	0.1182	0.1210					
GLMQ-YY	0.1335	0.1321	0.1335	0.1255	0.1247	0.0133				
GLMQ-RJ	0.1347	0.1366	0.1347	0.1365	0.1301	0.0366	0.0235			
FDNL	0.1325	0.1307	0.1325	0.1205	0.1196	0.0082	0.0153	0.0387		
GETU	0.1321	0.1302	0.1321	0.1200	0.1216	0.0016	0.0139	0.0372	0.0087	

The genetic distance among the 11 haplotypes of the mt*COI* gene of *C. loewiana* ranged from 0 to 0.3303 (Table 5), with an average of 0.1107. Among them, the genetic distance between H8 and other haplotypes was particularly high, ranging from 0.1744 to 0.3303. The genetic distance between the 5 geographic populations of this species ranged from 0.0016 to 0.0387 (Table 6), with an average of 0.0197. The genetic relationship between the GLBM population from Banma County and between GETU and FDNL populations was closer, followed by GETU and FDNL, and GLMQ-YY and GLMQ-RJ populations. However, the genetic distance between the GLMQ-YY and GLMQ-RJ populations differed significantly from other populations.

### Demographic Analysis

**Fig. 2.**
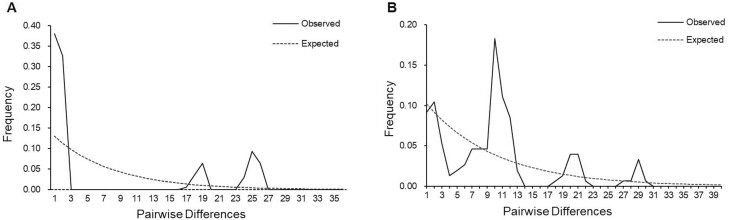
Mismatch distribution analysis of 10 populations of *T. angustipennis* and *C. loewiana* based on mtDNA *COI* gene. A and B represent the mismatch distribution analysis results of 5 populations of *T. angustipennis* and 5 populations of *C. loewiana*, respectively. This picture was made by Li-Jun zhang.

The 5 geographic populations of *T. angustipennis* and *C. loewiana* were analyzed based on mt*COI* gene sequences, and Tajima’s *D* values were −0.86939 and −0.54369, respectively. These values suggest significant differences among populations, which was evident from the results of the neutral test. When the geographical population of each species was analyzed, the various groups of the 2 species did not reach the significance level. A Tajima’s *D* value close to zero indicates a stable population, while a negative *D* value indicates that an abundance of rare alleles (such as low-frequency haplotypes detected in the population) may be due to a recent population expansion. Therefore, the results indicated that both pest populations have experienced significant recent population expansion. The mismatch distribution curves for the entire population of *T. angustipennis* (Fig. 2A) and *C. loewiana* (Fig. 2B) showed multiple peaks, although only the main peak was prominent (Fig. 2). This suggests that the geographical populations of both species have recently underwent or are currently undergoing significant population expansion events. The effective population size of the species has changed over time.

### Phylogenetic Analysis

**Fig. 3.**
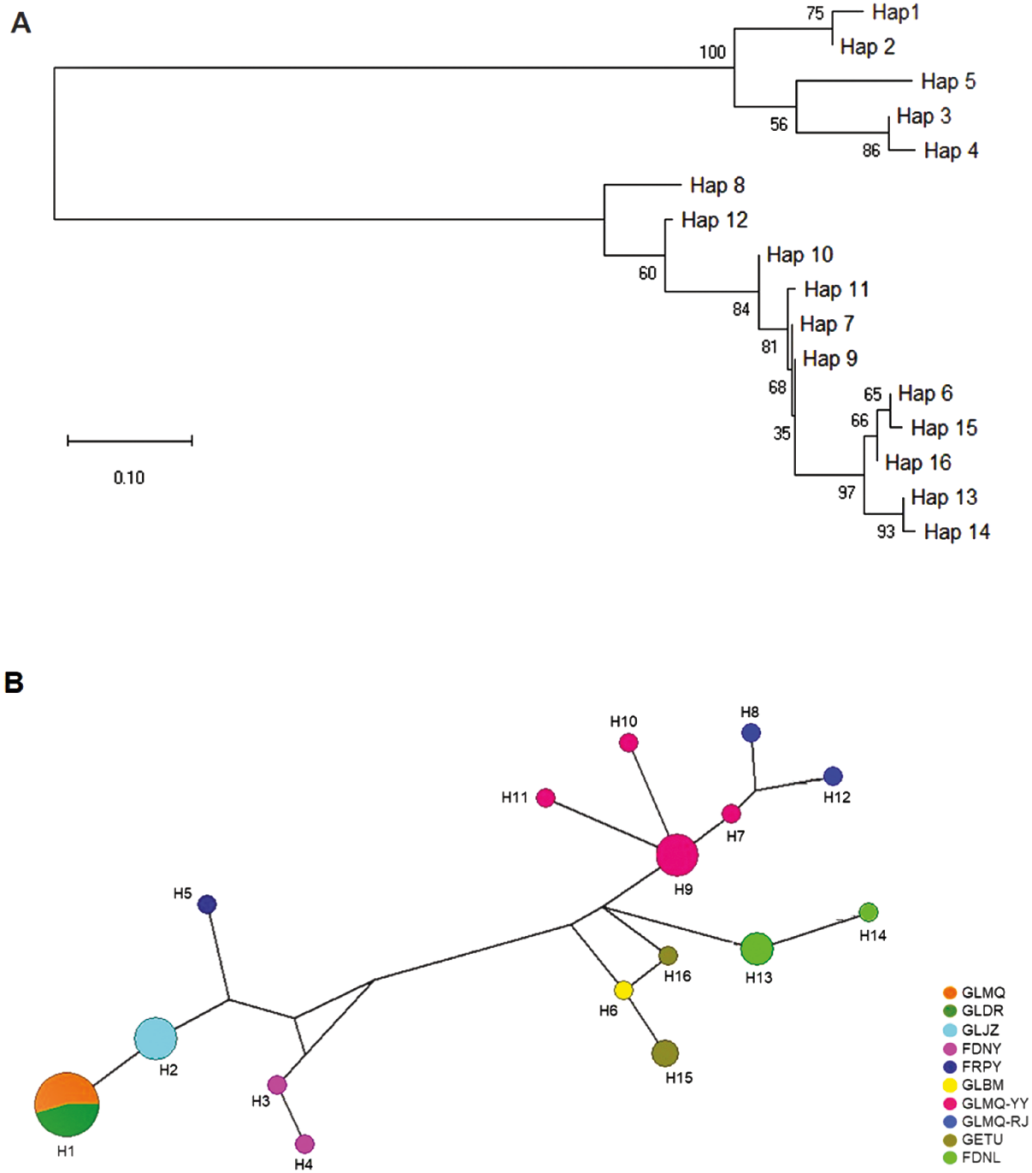
Phylogenetic tree A) and haplotype network B) of mtDNA *COI* haplotypes of *T. angustipennis* and *C. loewiana*. In haplotype network, colors represent different sampling sites. Area of the circles represented the number of observed individuals showing such haplotype. This image was created by Li-Jun Zhang.

Neighbor-joining phylogenetic trees of different haplotypes (Fig. 3A) and haplotype mediator networks (Fig. 3B) were constructed using mtDNA *COI* sequences of 2 species. The topological structure clearly distinguished the haplotypes of the 2 species, dividing the phylogenetic tree into 2 distinct branches overall. Among them, the first branch contained 5 haplotypes (H1–H5) representing *T. angustipennis*, while the second branch contained 11 haplotypes (H6–H16) representing *C. loewiana*. The NJ analysis revealed that different populations from the same geographic region of *T. angustipennis* and *C. loewiana* did not clearly separate during clustering. This indicates that individuals from different populations within the same geographic region may cluster together before the haplotypes within the same population, suggesting a parallel distribution relationship. In contrast, populations from different geographic regions showed obvious branches during clustering.

In the first strand, 2 haplotypes from China (H1, H2) clustered together, while 3 haplotypes from outside China formed a distinct clade. This indicates a close genetic relationship between the GLDR, GLMQ, and GLJZ populations, whereas the genetic relationship between the FRPY and FDNY populations is more distant. In the NJ tree of *C. loewiana*, the 2 haplotypes from the FDNL population clustered together, and haplotypes from the GLMQ-YY and GLMQ-RJ populations exhibited a relatively close genetic evolutionary relationship. Interestingly, the H6 haplotypes from the GLBM populations in China clustered with haplotypes H15 and H16 from the GETU populations outside China, showing a close genetic relationship ((H6, H15), H16). According to the clustering results from the haplotype intermediate network diagram (Fig. 3B), 2 distinct branches were formed, H1–H5 (haplotype of *T. angustipennis*) and H6–H16 (haplotype of *C. loewiana*), with the haplotype distributions of the 2 populations being relatively clustered. This is consistent with the evolutionary tree analysis results.

## Discussion

### Applicability of *COI* Barcodes for Molecular Identification of Two Species

The integration of molecular methods with morphological identification enhances the comprehensive and accurate identification of insect species, especially in complex species scenarios or with similar species (Ovtshinnikova and Ovchinnikov 2016, David et al. 2020). This involves the entire process from sample collection and preservation, morphological identification, selection of molecular markers, DNA extraction and PCR amplification, sequencing and sequence analysis, to the construction of phylogenetic trees and species identification, validation, and structural interpretation. This approach is not only applicable for confirming known species but also helps in discovering new species and studying evolutionary relationships and ecological adaptability among species. During field surveys, initial insect identification relies on traditional morphological methods. When traditional taxonomy is hindered, relevant samples are preserved for subsequent molecular analysis. At this point, DNA technology offers significant advantages by overcoming the limitations of conventional insect identification methods, showcasing its potential for widespread scientific applications (Prabhulinga et al. 2021, Saher et al. 2021).

Based on characteristic data from traditional classification, DNA technology provides a simple and rapid method for insect identification and a novel approach for taxonomists to discover new species (Wei et al. 2023). However, the DNA barcodes of *COI* genes for *T. angustipennis* and *C. loewiana* have rarely been reported in this study area, and many species of Tephritidae have similar appearances. The limited availability of morphological analysis indices makes accurate species determination through morphological observation of eggs, larvae, and adults difficult (Korneyev 2021, Mazzon et al. 2021, Jennings 2023). DNA barcoding enables quick and accurate identification of these 2 pests, irrespective of their developmental stage (Rathnayake et al. 2023). Therefore, this study utilizes taxonomic characteristics (such as shape, size, and color) for initial identification, followed by DNA barcoding identification methods for rapid identification and subsequent analysis.

Ultimately, 37 *COI* gene sequences were obtained in this study, and the mitochondrial DNA control region sequences of the 2 species of *Aster* pests were rich in *A* + *T*, showing an obvious base shift consistent with the base composition of typical insect mtDNA *COI* sequence (Simon et al. 1994). The nucleotide polymorphic site variation types of the 2 species of Tephritidae in different geographical populations were switched, consistent with the rule that the conversion was greater than the transposition in the taxonomic order with closer relatives, while the reverse is true for the taxonomic orders with more distant relatives (Simon et al. 1994). Simultaneously, both *R* values were equal to 2, indicating that the mtDNA *COI* gene sequence mutations of the 2 species had not reached a saturation state and could be used to study genetic diversity (Knight et al. 1993). The NJ tree branches for the *COI* sequence of the 2 species showed robust support, allowing clear distinction between them. Therefore, the *COI* gene segment can be used to identify these 2 species of Tephritidae, and can be preferentially selected as an effective molecular marker for the identification of Tephritidae, consistent with previous reports on the use of mt*COI* barcodes for identifying Tephritidae species (Wang Bixin and Lianfang 2002, Korneyev 2004, 2021, Jiang 2015, Chen 2017). However, the *COI* gene has a low success rate in the amplifying some Tephritidae samples. Even with repeated amplification attempts, sequences of all individuals cannot always be successfully amplified, or the sequencing results may show overlapping peaks, making the sequences unusable for subsequent analysis. Therefore, technical optimization throughout the entire process from sample collection to PCR amplification is essential. Additionally, if some applicable nuclear genes can be used as a supplement to the *COI* barcode, it needs to be validated. These could be used in lower-order units such as subgenus or subspecies of Tephritidae to improve amplification and identification success rate, which necessitates confirmation in larger future studies.

### Genetic Distance and Phylogenetic Relationship

This study utilized partial sequence of the mt*COI* gene, combined with *COI* sequences of the 2 pests published in NCBI, to understand the genetic relationships among various geographic populations of *T. angustipennis* and *C. loewiana* from different regions and with reported populations abroad, aiming to explore the possibility of host transfer and determine their phylogenetic history. We examined the genetic relationships and phylogenetic evolution of 5 populations of *T. angustipennis* and 5 populations of *C. loewiana* from TRSR, China. Previous studies have shown that 98% of insect species identified by morphological classification exhibit differences greater than 2% in mt*COI* gene sequences; thus, 2% is generally considered a threshold for species distinction (Hebert et al. 2003). Our study found that the genetic distance between *T. angustipennis* populations in the TRSR was 0.0016, less than 2%, indicating a closely related genetic relationship with no obvious genetic differentiation among the populations. These findings underscore the reliability of the genetic distance and phylogenetic relationships observed between these 2 pests in the TRSR region.

Except for the GLMQ-YY and GLBM populations (0.0133), the genetic distance of other populations ranged from 0.0235 to 0.0366, indicating that the GLMQ-RJ population exhibited significant genetic differentiation from the GLBM and GLMQ-YY populations. The NJ molecular phylogenetic tree revealed that the 5 populations of *T. angustipennis* were not completely separated, whereas those of *C. loewiana* were more dispersed. Overall, different populations of *T. angustipennis* and *C. loewiana* from the same geographical region did not branch significantly during clustering, meaning individuals from different populations in the same region may cluster together before haploids from the same population. This indicates a parallel distribution relationship, suggesting that genetic differentiation among populations in the same geographic region is not pronounced. This finding contradicts the conclusion of nonparallel morphological differentiation of new host plants after colonization in the host transfer study on *Tephritis conura* (Nilsson et al. 2022). However, populations from different geographical regions branched significantly in the cluster, which may be attributed to greater geographical isolation leading to more obvious genetic differentiation. Consequently, we infer that these 2 species of Tephritidae may have diffused recently, and due to the short history, the accumulation of mutations in the mitochondrial genome is insufficient to result in large genetic differences. However, the limited sample sizes from populations outside China severely restrict the available information for analysis and statistical purposes. Therefore, sustained research attention to this pest is essential, especially beyond China is crucial.

### Genetic Diversity

Genetic diversity is the foundation of biodiversity and guarantees species evolution. In this study, 5 haplotypes were identified in the mtDNA *COI* gene of 5 *T. angustipennis* populations, including 2 haplotypes in the TRSR populations, and 1 shared by GLDR and GLMQ (H1). In contrast, 11 haplotypes were found in 5 *C. loewiana* populations, with 7 haplotypes in the TRSR populations and no haplotypes shared by all populations. These findings indicate greater genetic diversity in *C. loewiana* than in *T. angustipennis*, and mtDNA of each geographic populations in the TRSR district exhibits both gene exchange and genetic differentiation (Bondaryuk et al. 2022).

Among the 5 haplotypes of *T. angustipennis*, H1 is the most widely distributed and dominant. This prevalence may be due to its primitive nature and superior ecological adaptability. There are no multiple invasion sources of *T. angustipennis*. Conversely, the haplotype of *C. loewiana* do not exhibit dominance over those of *T. angustipennis*, indicating the possibility of multiple invasion sources of *C. loewiana* in TRSR. In both populations, except for H1, all haplotypes are private, indicating a biologically suitable environment that minimizes interspecies gene exchange and maintain stable genetic diversity (Fang et al. 2022, Li et al. 2022, Wang et al. 2023). High haplotype diversity in the total populations of *T. angustipennis* and *C. loewiana* indicates high polymorphism in the mt*COI* gene. Among *C. loewiana* populations, the GLMQ-RJ population is the most polymorphic. The *F*_*st*_ and *N*_*m*_ values of the 2 populations were analyzed using mitochondrial genes. The *F*_*st*_ were 0.73226 and 0.53507, respectively, and the *N*_*m*_ values were 0.18 and 0.43, indicating high levels of gene exchange between different populations of the 2 species, with some genetic differentiation.

### Genetic Differentiation

AMOVA demonstrated that the genetic variation between populations of the 2 species mainly results from geographical isolation on the QTP or potentially from factors like air diffusion (Wei et al. 2021, Zhao et al. 2021). An extremely low Tajima’s *D* value from the neutral test suggests recent rapid population outbreaks and expansions in both *T. angustipennis* and *C. loewiana* populations (Sun et al. 2011, Pflugbeil et al. 2021). Combining these findings with population misallocation analysis, it is inferred that populations of *T. angustipennis* and *C. loewiana* in TRSR have recently undergone or are currently experiencing notable population expansions, correlating with increased severity of *Aster* infestation and expanding occurrence annually. Adaptive evolution of *Tephritis* species toward host plants has led to genetic differentiation among populations (Liu 2021), but reproductive isolation has not yet occurred. Considering the widespread impact of plants on species interactions between phytophagous insects and parasitoid wasps, plant-mediated trophic interactions may contribute significantly to species diversity. Further studies are warranted to explore the relationship between *Aster* plants and pests in TRSR.

The haplotype evolutionary tree and intermediary network diagram revealed distinct geographical clustering of each haplotype within the 2 species based on their distribution. However, exceptions were noted, such as the dispersion of the GLBM population of *C. loewiana* in the TRSR with the GETU population outside TRSR, and the clustering of haplotype 6 from TRSR populations with haplotypes 15 and 16 from German populations. Given the limited flight capability of these small flies, long-distance spread within TRSR or even between different countries likely depends on seedling transport, wind dispersion, and human activities, potentially facilitating genetic exchange. Meanwhile, haplotype clustering indicates some similarity among these haplotypes; although their distribution in different geographic regions, they may share common ancestors or ancient genetic connections, which are crucial factors shaping their distribution. These findings also highlight their adaptability to Asteraceae plants and survival strategies under specific environmental conditions.

In conclusion, this study integrates morphological preliminary identification with *COI* barcode labeling, providing important insights for species identification of fruit flies on *Aster*. It enhances understanding of the genetic structure, evolutionary history, and ecological adaptation of these populations. It also offers important scientific basis and guidance for biodiversity research, pest management, and ecological conservation. Specifically, studying the genetic structure of both pest populations can provide the following insights for pest management: (i) genetic structure analysis can inform resistance management strategies, aiding in selecting of the most effective pesticide types and usage strategies to prevent further resistance spread. (ii) Monitoring and predicting population dynamics of fruit fly outbreaks can be achieved through regular genetic structure monitoring, facilitating geographical distribution and change models to predict migration and spread paths for timely control measures. (iii) Environmental adaptability analysis can evaluate how different genetic types of fruit flies respond to environmental changes, predicting their behavior under climate change or new environmental conditions. This assists in devising flexible and effective management strategies. (iv) Precise biological control strategies can be developed by understanding resistance differences among different genetic types of fruit flies to predators or parasites. This optimization enhances the effectiveness of biological control measures by selecting suitable natural enemies or parasitoids. (v) Population monitoring and management: regular monitoring of genetic structure changes in fruit fly populations helps assess the effectiveness of management measures and adjust strategies promptly to cope with population dynamics. Future analysis should integrate additional molecular markers and study more individuals to elucidate the drivers of genetic variation and population distribution patterns of *T. angustipennis* and *C. loewiana,* as well as research on the interactions between pests and host plants is needed to confirm and deepen the understanding of relationships among these haplotypes and their associations with host plants. This comprehensive approach is crucial for formulating effective biological control strategies against these significant pests harming *Aster* inflorescences in the TRSR.

## Supplementary Material

ieae075_suppl_Supplementary_Material
